# Living with Bears in Prahova Valley, Romania: An Integrative Analysis

**DOI:** 10.3390/ani14040587

**Published:** 2024-02-10

**Authors:** Alina-Lucia Cimpoca, Mircea Voiculescu, Remus Creţan, Sorina Voiculescu, Ana-Neli Ianăş

**Affiliations:** Department of Geography, West University of Timişoara, Bdul Vasile Pârvan, 4, 300223 Timişoara, Romania; lucia.cimpoca@e-uvt.ro (A.-L.C.); remus.cretan@e-uvt.ro (R.C.); sorina.voiculescu@e-uvt.ro (S.V.); ana.ianas@e-uvt.ro (A.-N.I.)

**Keywords:** posthumanism, tourist resorts, human–bear interactions, bear zoonym, souvenirs, Prahova Valley, Romania

## Abstract

**Simple Summary:**

This research focuses on the perception of residents and tourists from the tourist resorts in Prahova Valley, Romania, on human–bear interactions. As a foundation for an integrative analysis of the presence of bears in this space, we used questionnaires, interviews, mass media, and the local toponymy including bear-related names and souvenirs that embody the bear. Our results have highlighted the beginning of the coexistence between humans and bears and the seeds of a posthumanist vision.

**Abstract:**

Our research focuses on a complex and integrative analysis of bear presence in four tourist resorts in Prahova Valley, Romania: Sinaia, Bușteni, Azuga and Predeal. Employing innovative mixed methods, including questionnaires, interviews, newspaper analysis, and consideration of the local toponymy, including bear-related names and souvenirs, we aim to highlight the extent to which a posthumanist attitude is evident in the region. The sustained appearance of bears is attributed to habitat invasion through deforestation, road construction, residential neighborhoods, and tourist infrastructure. Ambiguity arises from the presence of food sources and voluntary feeding both by locals and tourists. The mass media initially heightened fear and panic during the onset of human–bear interactions but later adopted a more tolerant tone regarding the bear’s presence in tourist resorts, reflecting an openness to the posthumanist approach in Prahova Valley. That is why locals express fear and concern about bear encounters, advocating for a clear separation between animal and human spaces. Tourists exhibit attitudes ranging from unconscious appreciation to ambivalence, often contributing to the problem through practices such as feeding bears for fun. The use of bear-related names for tourist establishments is identified as anthropocentric, despite their appeal for attracting tourists. Souvenir sales, through increasing socio-economic value and contributing to tourist experiences, are also recognized as anthropocentric. However, souvenirs can provide elements of support for bear conservation efforts and the equal consideration of human and non-human entities. This study concludes that a successful adaptive coexistence requires a posthumanist vision, overcoming anthropocentrism in a landscape altered by human activities, supported by bear management programs in Bucegi Natural Park, and conservation efforts in Prahova Valley in a landscape altered by people.

## 1. Introduction

Bear species are considered elements of wild fauna, large carnivores [1,2,3], wildlife tourism [4,5], and emblematic species worldwide [6,7]. The spatial expansion of human settlements and various activities have influenced the shrinking habitats of wild fauna [8,9,10]. In this context, the interaction between humans and wild animals, often at the border of protected areas, has increased, with dire consequences on both sides [11,12].

The most predominant factor with direct influence on biodiversity loss is the urbanization process through oversizing human settlements, which causes habitat fragmentation and landscape change [13,14,15]. In contrast, the fragmentation of forests also contributes to biodiversity loss and the fragmentation of habitats occupied by wild fauna [16,17,18]. Specifically, the different interactions between humans and bears have increased alarmingly, with economic and sociocultural impacts on humans [19,20], as well as impacts on the ecology and feeding behavior of bears [21,22]. Legal obstacles, such as those posed by the European Union, also interfere with traditional national regulations [23].

Romania managed bears as game animals that were hunted for trophies, under communist and post–communist regimes [24], whereas the European Union regarded them as protected species, which prohibited their hunting and aggravated the tolerance of the communities towards daily encounters with bears. Meanwhile, carnivore trophy hunting was banned in Romania in October 2016 [25,26]. Moreover, mountainous areas with a high degree of urbanization face frequent human–bear interactions (HBIs) [27]. These interactions are extremely intense near tourist resorts, where tourism affects wildlife [28,29], causing biological bear invasions [30,31].

The growing number of human–brown bear (*Ursus arctos*) interaction occasions in the Romanian Carpathian space have recently attracted the attention of geographic researchers. In two counties in the interior Carpathian Mountains of Transylvania, Stăncioiu et al. [32] highlighted the coexistence of humans and bears. Using statistical and network analysis, Pătru-Stupariu et al. [33] proposed a different approach to investigating human–wildlife interactions and perceptions of the local population. Another study was based on surveys applied in Prahova Valley to highlight the occurrence and triggers of human–wildlife interactions, with the brown bear being considered as a problematic species [34]. Based on 931 media articles, some authors analyzed how the mass media in Romania reflected HBIs [35]. On the other hand, several researchers evaluated the connectivity of the main species in the Romanian Carpathians, including the brown bear, and identified priority areas to ensure connectivity [36,37].

### Posthumanism in the Context of Human–Animal Interactions

Posthumanism is defined as an ethical stance, a moral concern regarding non-human beings [38,39], a postmodern philosophical discourse that takes into account “the ethics of speaking for and about non-human animals” [40]. Posthumanist theory started from the premise that human beings were considered separate from non-human entities [41,42], and human superiority would undergo a “deconstructive movement” in human–animal relationships [43]. In the posthumanist vision, humans lose their anthropocentric cultural dominance, and animals become significant actors in human–animal interaction contexts, ‘the human being reconfigured in posthuman convergence’ [44].

Perhaps that is why some authors envisioned an ethical-driven posthuman future, where humans make room for the non-human lives, especially wildlife [45]. Posthumanism regards humans as any natural species and not above nature [46], with coexistence being the only path to peaceful cohabitation between humans and wildlife. In the same vein, coexistence is defined as a path where humans and wildlife adapt through landscape sharing, and interactions between humans and wildlife are efficiently governed to the extent that the latter exists legally and socially, ensuring a tolerable level of risk [47].

Geography as a discipline has helped posthumanism become a key approach in the social sciences [48], thus highlighting the role of animal geography as an essential contribution to the posthuman pursuit of animal studies [46]. Humanist geographers have employed posthumanist theory to shift away from the dominant anthropocentric view, aiming for a better understanding of non-human entities based on “actions, emotions, and affects” [49]. Anthropocentrism poses challenges to the conservation process. Nature should not be separated from culture, meaning from humans and their activities. Human interests should not take precedence, dominating over non-human entities.

Perhaps that is why the concept of anthropocentrism has sparked many debates in the scientific community, not necessarily limited to geography.

Anthropocentrism has been defined “as the love of one’s own species and, secondly, as discriminating against other species” [50]. Callicott [51] speaks of human interest being given a hierarchically superior position compared to non-human entities, which are assimilated only to human needs, a perspective specific to environmental education thinking [52]. On the other hand, Kopnina [53] defines anthropocentrism as “a powerful explanation for society’s current environmental unsustainability and unethical treatment of nonhumans”. In the same context, Kopnina [54] mentions that “posthumanism exposes anthropocentrism as an attempt to ignore behavior in which humans focus on themselves at the expense of all other species”. He argues that anthropocentrism is an ethically misguided and impractical path. A successful coexistence must be based on the ethical consideration of non-human entities from a human perspective, not anthropocentrically, with humans as the sole beneficiaries of the opportunities provided by the environment in which they live [39].

Posthumanism counters anthropocentrism and relies on a process that diminishes “the destructive human/non-human divide in our enactment of the urban commons and the urban commoners” [55], emphasizing “respect, fear, and reverence for the rich and vital structure of planetary entanglement that is the biosphere and the Earth system”. This holds intrinsic value as the material source and envelope for human life [56], representing the only way to rethink and reshape the interaction between humans and non-humans [44].

The opening towards ethical posthumanism emerging in Prahova Valley represents a new analytical path, not just circumstantial but real. Here, residents and tourists, despite the contradictory uncertainties in understanding the presence of bears, attempt to establish relationships of coexistence, especially in the last 10–15 years. Our study aims to analyze the concept of living with bears, in other words, human–bear coexistence in a landscape strongly dominated by humans or in a human-modified landscape sense [12].

Therefore, to find a possible perspective on this coexistence, we addressed two questions: (i) Does human–bear coexistence exist in Prahova Valley? (ii) To what extent is the success of good coexistence in this area attributed to posthumanism?

## 2. Materials and Methods

### 2.1. Study Site

**Figure 1:**
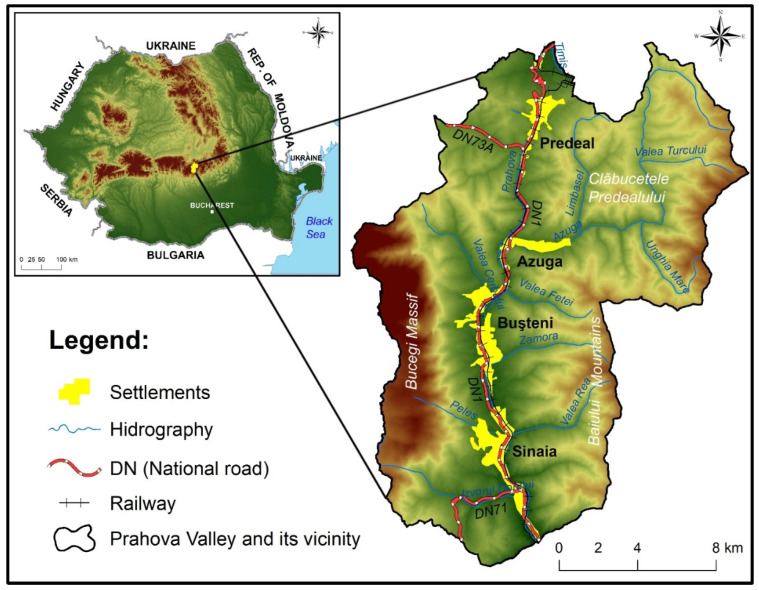
Location of the study area.

Prahova Valley is located between two important mountain massifs, the Bucegi Mountains (west) and the Baiului Mountains (east). It represents a heavily populated area with towns at an altitude of 850–1059 m: Sinaia (10,659 inhabitants), Buşteni (9122 inhabitants), Azuga (4511 inhabitants), and Predeal (4712 inhabitants) [57] (Figure 1).

The Bucegi Mountains have been proposed as a natural park since 1938. Much later, in 1990, the Bucegi Mountains were declared a natural park only for forested areas by an M.A.P.P.M. under order no. 7/27.01.1990. According to government decision no. 230/2003, the Bucegi Mountains are now a fully protected area. This natural park has an area of 32,663 hectares [58]. Mountain tourism is conducted throughout the year. The Bucegi Mountains presents a well-developed tourist infrastructure, and thus attracts numerous tourists. The settlements have a linear arrangement extending towards the mountainous area and clearing the forest. This development model resulted in bears descending into urban and peri-urban areas in search of food and their fear of humans being removed [34]. According to official statistics reported by the Administration of Bucegi Natural Park (BNP), the population of bears during the last 15 years has been 100. However, the number is higher, approximately 130–140, and the area can support no more than 30 or 40 bears based on the interviews conducted with the Dacia Hunting Association and Sinaia Hunting Association representatives.

This model of urban expansion, with massive encroachment of the mountain space and the transformation of the streets or ski slopes into non-stop entrance lanes, includes factors influencing the access of brown bears within human settlements. Therefore, man and bear invade each other’s habitat, potentially resulting in threats to both parties (man destroys the bear’s habitat, and the bear causes damage or poses a threat to man) [11].

### 2.2. The Integrative Methodology

The method that we used in our study is novel as it has not been carried out by other studies in human–animal interactions studies. We entitled this method as an integrative methodology for human–animal interactions because we used both qualitative and quantitative data taken from people living in the studied area, a newspaper analysis, as well as tourist souvenirs and toponymic data, namely, bear zoonym data related to bears. These data were supplemented with field observations.

**Figure 2:**
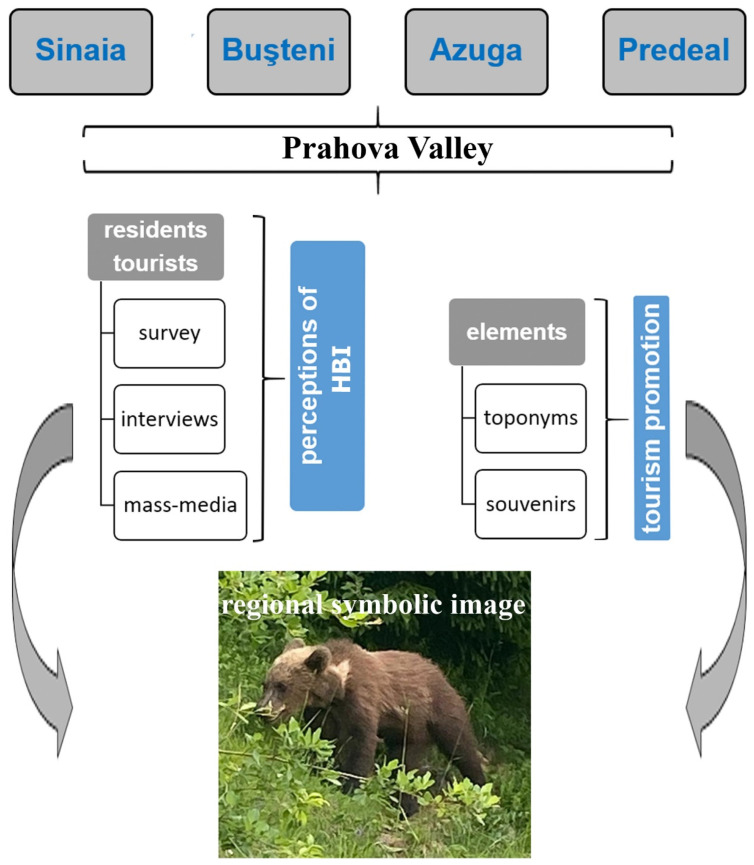
A flow chart of the field methods (detailed in the text).

Our study combines the analysis of local narratives regarding HBI with a scientific design called the generic triangulation method, with the priority of ensuring the validity of the qualitative research [59,60]. The qualitative aspects were based on surveys and interviews conducted under the impact of the pandemic and a triangulated mass media analysis to observe common themes emerging in the societal response to human–bear interactions. We also used bear zoonym analysis and bear souvenirs to give a new, integrative, dimension to our analysis. All these approaches revealed, in the sense shown by Dorresteijn et al. [61], the different perceptions of residents and tourists that define the coexistence of humans and bears in Prahova Valley, as well as the forms of local management. A flow chart of the field methodology applied is presented in Figure 2.

### 2.3. Survey and Semi-Structured Interview Design

Wildlife analysis successfully used the survey to evaluate people’s attitudes and perceptions of HBIs [62,63]. Due to pandemic conditions, we decided in July and August 2021 that the survey should be distributed online, through social media platforms (Facebook and Instagram) [64,65] and by e-mail [66,67], targeting accommodation service providers. We also obtained answers on-site in September 2021. The survey comprised 24 questions and was applied to a sample of 210 respondents evenly distributed among the four investigated tourist resorts: Buşteni, Sinaia, Azuga, and Predeal. Our survey was conducted with residents (*n* = 90) and tourists (*n* = 120), and all participants verbally consented to the survey. The survey characteristics are presented in Table S1a.

We used a semi-structured interview [61] targeting three major themes: (i) the causes of the presence of bears in the tourist resorts of Prahova Valley, (ii) proposed local policies to remove bears from communities and reduce human–bear interactions, and (iii) the involvement of authorities in preventing these interactions [66,68]. We interviewed 36 people, with 9 people per resort to ensure a uniform distribution of the interviews, and the interviews lasted 30 to 40 min. Our interviews were conducted with several local actors (Table S1b). We also contacted members of the town halls in tourist resorts, but they could not be interviewed. In January 2022, we conducted twelve telephone interviews, as has been carried out in other studies [66,69] efficiently for data collection [70,71], and we held six meetings using the Google Meet application. After the relaxation of the pandemic restrictions during the first half of April 2022, between 1 July and 1 August 2022 we also conducted twelve walking interviews in natural surroundings, as has been performed in other studies [72,73], and six sit-down interviews with people who preferred the comfort of home [74,75]. All interviews were recorded and later translated into English, and all participants verbally consented to the interview.

### 2.4. Mass Media—First Source of Local Information

In general, HBIs have attracted the attention of the mass media, which has alerted the public and professionals to this controversy [76]. Mass media, as a qualitative method, are the first source of local information [77], and the means used and the frequency of the appearance of news play an essential role in the degree of people’s perception of human–wildlife coexistence [78,79]. Mass media that present HBIs and amplify the perception of risk can cause adverse reactions towards bears and their conservation forms, having an alarming character [66,80], mainly when sensational and terrifying news headlines cultivate fear and mistrust [81]. However, objective information can promote safety and unbiased information on HBIs [82]. Seven national and regional newspapers and multimedia platforms were chosen as news archives (Table S1c), and the information was selected based on the representativeness and usefulness of the information about human–bear interactions. To identify relevant news, we used the keywords relevant to our study [35], namely, bears, Prahova Valley, and HBIs. The variables used were the newspaper’s name, general specialization of the newspaper, territorial distribution, periodicity, general policy of the newspaper, and the number of news/date of the news.

### 2.5. Analysis of Toponyms and Souvenirs

The analysis of local toponymy including animal-related names, in this case bear-related names, is essential for geographical sciences [83], representing a suitable method for tracking the presence of the brown bear in the study area over time. Animal names given to places are stable indicators of heritage and bio-cultural diversity [84]. To identify them, we consulted topographical and geographic maps [85] and made field documentation.

Souvenirs are an important component of the tourist experience, a moment of life that can be remembered [86], playing a role in tourism development [87]. To highlight the motivation for the purchase and the role of souvenirs in cultivating the tourist experience, we used 52 semi-structured interviews (Table S1d) with both souvenir sellers (24 people) and tourist consumers (28 tourists) [88], as well as personal observations. The interviews took place in two locations dedicated to the sale of souvenirs: the alley to the Peleş Castle in Sinaia and on DN1 between the Sinaia and Buşteni tourist resorts. These areas are the most densely populated in terms of tourists and purveyors of souvenirs.

### 2.6. Data Analysis

We used two different scales for explanatory variables from our database [89], dichotomous and trichotomous, for the evaluation of the presence of bears in Prahova Valley, the attitude of the respondents towards the bears in terms of perceptions of fear, the attitude towards the feeding of bears, and the role of mass media in awakening the emotions and attitudes of the respondents during the HBI.

To evaluate personal protection measures to avoid bear encounters and degree of perceived risk in possible bear encounters, we used a 5-point Likert scale [16,90]: 1—very low risk, 2—low risk, 3—medium risk, 4—high risk, and 5—very high risk. All responses of the respondents highlighted the possible management measures and tolerance or inadmissibility of the bears in Prahova Valley.

The interpretation of all qualitative data was based on Bryman’s methodology [91]. All the data were transcribed in Romanian. Codes of the direct quotes of the respondents and those found in the press were selected. The major themes emerging from the analysis were determined following the societal response to man’s interaction with the brown bear.

## 3. Results

### 3.1. Main Problem of Presence of Bears in Prahova Valley: How Narratives of Residents and Tourists Articulate Different Responses, in Terms of Perception

The presence of bears in Prahova Valley has been evident for the last 30 years. The locals interviewed mentioned that bears were not encountered in the 1960s or 1970s: I have never seen a bear in Sinaia and its surroundings. I lived in these places in the 1970s when I attended ski school (a 55-year-old woman, Sinaia) or I never saw a bear when I was a child or in my adult life in Sinaia. Now I see them near Peleș Castle. Even though I love them, I’m afraid of them (a 55-year-old woman, Sinaia). Recently, a series of press articles emphasized the localization of the appearance of bears in the tourist resorts of Prahova Valley (Table S2).

**Figure 3:**
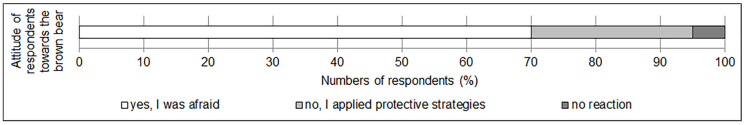
Attitude of respondents who experienced encounters with bears.

Most respondents (35 residents) unanimously answered that they had experienced encounters with bears, being afraid. Others applied protective strategies: avoiding the areas near the forest at nightfall, leaving baskets and garbage bags behind closed gates, and avoiding the streets at night except for heading to or from work (Figure 3). In this sense, the opinions of the interviewees were that the bear represents a danger for the physical integrity of people (a 55-year-old man, Sinaia) and people should not interact with wild animals that should stay in their natural environment, away from people (an 18–25-year-old man, Braşov). Other locals believe that there are many tourists who come to this area just to see the bears (a 36–45-year-old woman, Bușteni).

**Figure 4:**
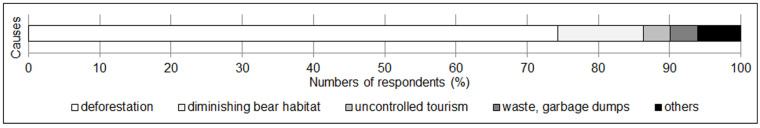
Main causes of HBIs.

Dumpsters and landfills, as well as uninformed tourist activities, such as feeding of the bears, attract the animals, increasing fear among locals (Table S3). They feel neglected in this fight because they believe that protecting the bears is prioritized over protecting the local people. Deforestation, diminishing bear habitats, uncontrolled tourism, waste and garbage dumps, and even climate change are the main causes of HBIs (Figure 4).

**Figure 5:**
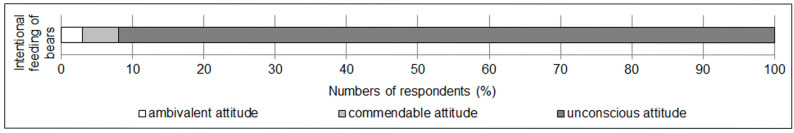
Intentional feeding of bears.

The practice of feeding bears for tourist purposes is common in Prahova Valley. However, the respondents’ perceptions varied considerably, from unconscious and appreciative attitudes to ambivalence (Figure 5). Some locals treat the bears as pets, leaving food for them: The bears are fed by tourists and locals. I know a family that, out of pity, fed a bear cub every evening in front of the house. The cub grew up and became a female who now has two cubs, and together they come in front of my neighbor’s house, as she used to for years. My neighbors want to evict her, and they asked the authorities for help, but no chance. The bear has gotten used to that place and knows that there is food there (a 55-year-old woman, Sinaia).

**Figure 6:**
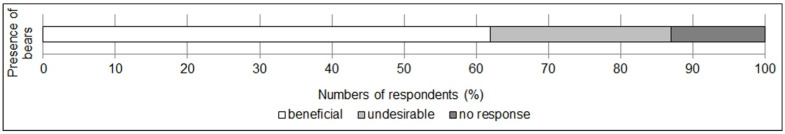
Opinions of respondents about the presence of bears.

The information obtained from the press was validated using the results of survey, where 62% of respondents thought bear presence boosted tourism. However, tourists held ambivalent opinions about the presence of bears (Figure 6). Some interviewees said the following: The bear is a wild and dangerous animal, and their presence in populated areas decreases the number of tourists (an 18–25-year-old woman, Bucharest); I think that sometimes the presence of bears is an opportunity to attract tourists because they are not considered a threat (an 18–25-year-old woman, Sibiu); the abundance of bears is a danger to both people and animals (an 18–25-year-old man, Lugoj); the presence of bears in populated areas is a risk factor. The population needs to be trained on what to do and what not to do when they see a bear. An optional course taught to students and beyond would be beneficial (a 46–55-year-old man, Buzău).

### 3.2. Associated Risk Perceived by Locals and Tourists

**Figure 7:**
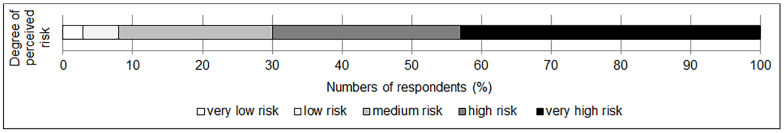
Degree of perceived risk in possible encounters with bears.

Based on the Likert numerical scale with variables ranging from 1 to 5, participants indicated the degree of perceived risk in bear encounters (Figure 7).

The results indicated that the subjects were aware of this danger, 68.6% (of the total respondents, *n* = 144) considering that the presence of bears near human settlements and even inside them represents a very high and high risk for human communities. Of all respondents, 21.4% of the total respondents (*n* = 45) believed that the presence of bears represents a medium risk. At the opposite pole, 10% of the total respondents (*n* = 21) believe that the presence of bears represents a low risk and very low risk, and the arguments are based on the idea that bears living near human communities develop an attachment behavior towards them, most often influenced by food.

### 3.3. Bear Management from a Local Authority Perspective

The representative of Bucegi Natural Park interviewed in this study mentioned that many bears lived in the Prahova Valley area 15–20 years ago. Currently, there are 130–140 bears, three times more than the carrying capacity of the territory. From the interviews conducted, we learned that the private administrators of the hunting funds were no longer delivering complementary food in the late autumn period, which is extremely necessary for bears after hibernation. This season is also the period in which the HBIs are recorded because the bears come down to the resorts in search of food.

**Figure 8:**
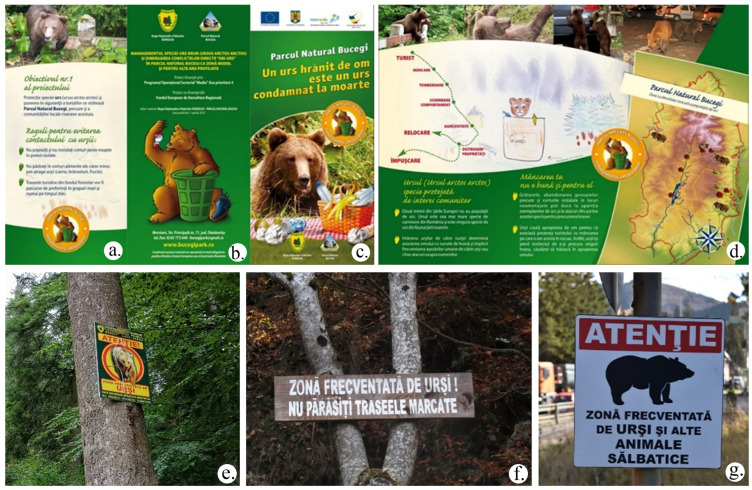
Leaflet of the project “Management of the brown bear species and the reduction of direct HBI in the Bucegi Natural Park as a model area for other protected areas’ [92]: rules for avoiding contact with bears (**a**); the bear (*Ursus arctos*) protected species of community interest (**b**); your food is not good for him (**c**); tourist–food–dumpsters–behavior change–aggression–destruction (**d**); Caution! Bears in the area. Do not leave the marked trails (**e**,**f**), and Caution! Bears and other wild animals in the area (**g**). (photos by Voiculescu Mircea).

Due to frequent human–bear interactions, in 2009 the project Management of brown bear species and the reduction of direct HBI in the Bucegi Natural Park as a model area and for other protected areas was initiated, which aimed to analyze the behavior of bears and promote awareness among locals and tourists. The slogan of this project was A bear fed by humans is a bear sentenced to death [92] (Figure 8a–d).

On this topic, we learned from interviews about the tragic story of Max Ursul, heavily publicized in the media, which was a bear that was fed sweets by tourists, fell ill with diabetes, and went blind. Other interviewees stated that he was brought in chains to the entrance to the alley leading to the Peleș Palace in Sinaia, by the abuser or owner. He lived off the money he received from tourists taking pictures with Max. At night he kept him in a pig pen, in a rented house across the road from our house. He gave him beer to calm him down. If he became aggressive, he was sprinkled with pepper in his nose… He was taken by the authorities after nine years of torture and taken to the bear sanctuary in Zărneşti. I don’t know why I didn’t take a stand then (a >55-year-old woman, Sinaia).

BNP representatives mentioned that the administration has carried out actions to inform tourists, and rangers go to the field every weekend to inform the public about possible HBIs and also to remove bears from tourist routes (a 36–45-year-old man, Bușteni). Since tourists are only protected during weekend tourism, we consider this shortcoming as a limiting factor for long-term tourism. These public information services intensified over time when there were major problems in conflict management. However, during our travels on the tourist trails, we did not encounter the rangers that the BNP representatives were talking about.

Cabin owners also advise tourists to stick to the marked trails, avoid feeding bears, and refrain from leaving garbage behind. It is important to note that bears do not eat the same food as humans.

### 3.4. Bear Management from a Respondent’s Perspective

All interviewees indicated that there are informative or local warning signs (see Figure 8e–g) about bears in the area, visiting rules, and warning messages strategically placed in key tourist spots in resorts and on trails in the following regions: the Peleș Castle in Sinaia, the Howling Waterfall and the Cantacuzino Castle in Buşteni, the ski slopes in Sinaia, Buşteni, and Predeal, and the areas with cable cars in Sinaia and Buşteni.

On the other hand, the locals interviewed mentioned I wouldn’t say that the bears come down to the community, but that they are chased out of the forest by the community (a >55-year-old man, Predeal), and some of those interviewed propose measures to remove bears from communities and reduce HBIs, creating sanctuaries where bears can be monitored, which is a financially expensive solution. Therefore, the same interviewee proposed donating 2% of his salary to the authorities that administer these sanctuaries (a >55-year-old man, Sinaia). Another respondent stated, dumpsters encapsulated and sealed in the ground exist, but they are useless if they are not collected in time, with bears having access to the waste that remains outside … there is no timely monitoring of waste collection by sanitation teams (a 26–35-year man, Bucharest).

In addition to the acceptance and habituation of the local residents to the brown bear, we learned from the local press about imminent and extreme situations that generate fear or even terror among the residents upon encountering a bear (Table S4). The key terms that characterized the articles were fear, terror, panic, and attack.

**Figure 9:**
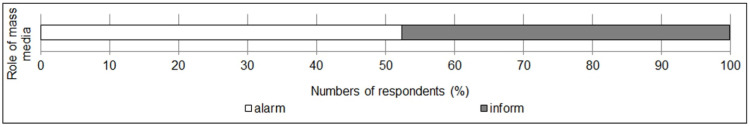
Role of mass media in providing information to the population.

Most respondents condemned the mass media for failing to provide useful information to the population (Figure 9): They don’t look for informative news, they always look for sensational headlines (a >55-year-old man, Sinaia); even if specialists request the publication of educational reports, the information transmitted by administrations is truncated. They offer one point of view, and something else is published (a 36–45-year-old man, Bușteni).

**Figure 10:**
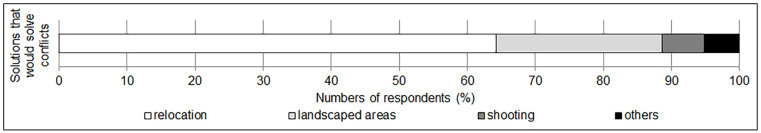
Solutions presented by respondents that would solve conflicts.

Maybe that is why some of the interviewed people believe that certain solutions (Figure 10) would solve conflicts: Shooting is the best solution (a 36–45-year-old man, Bușteni), or relocating to spaces where they can enjoy their own habitat, without having to share it with the people (a 36–40-year-old man, Predeal). Others, in contrast, supported carrying out information and awareness campaigns for tourists and locals (a >55-year-old man, Sinaia).

### 3.5. Toponyms and the Use of the Bear Name on the Firmament of Some Tourist Infrastructure Elements

**Figure 11:**
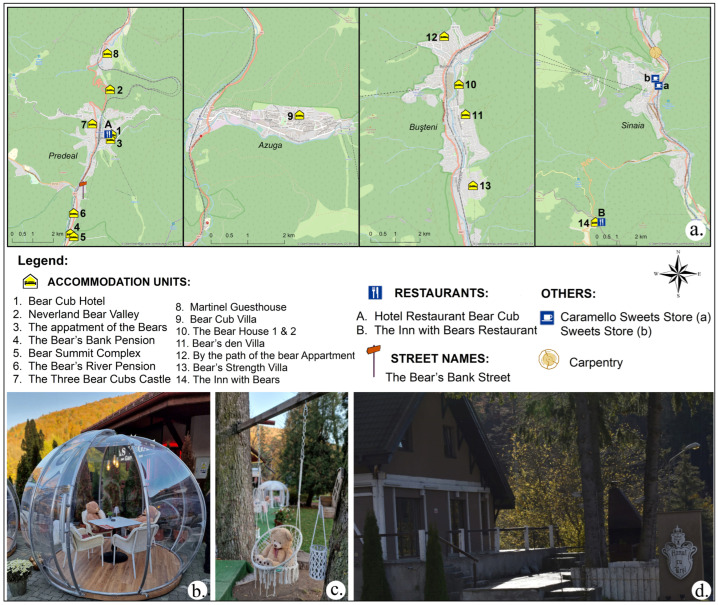
Bear toponyms (**a**), placement of teddy bears on the terraces of some confectioneries (**b**,**c**); and Bear’s Inn in Sinaia (**d**).

In the study area we identified bear-related names. These included names associated with tourist trails such as Happy Bear Trail on the eastern slope of the Bucegi Mountains, rivers such as Female Bear, Small Female Bear, which are tributaries of the Prahova River as it exits from Predeal towards Azuga, as well as landforms such as Bear Cave and Bear Keys in the Bucegi Mountains, and there is a Bear Bank Street in the Predeal resort (Figure 11a).

On the other hand, the incorporation of the word “bear” into the names of some accommodation places or restaurants, and strategically placing teddy bears on the terraces of select confectioneries, serves the purpose of tourism promotion (Figure 11b–d). This trend was also apparent in the interviews with statements such as we promote our guesthouse, the name attracts tourists (guesthouse administrator from Buşteni, a 36–45-year-old woman), and the teddy bears sitting on the chairs are meant to attract tourists (a 26–35-year-old woman, worker in the confectionery from Sinaia).

### 3.6. Souvenirs Representing Bears in Prahova Valley

**Figure 12:**
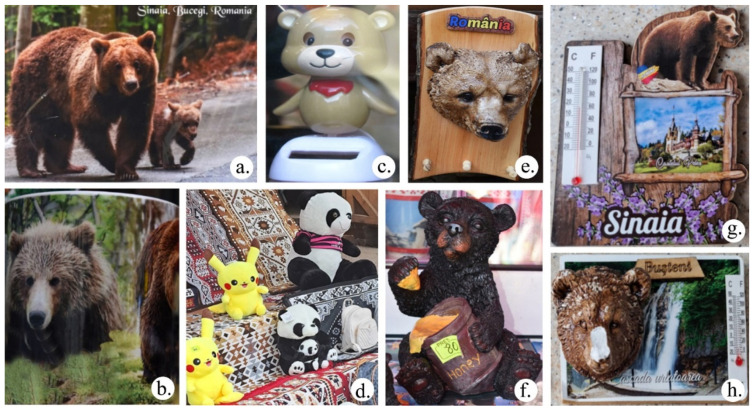
Souvenirs with bears sold in Prahova Valley: postcards (**a**); on cups (**b**); toys (**c**,**d**); wood sculptures (**e**,**f**); magnets (**g**,**h**).

The trade in bear-themed souvenirs is practiced by the people who live in the study area and it is a recent development: Since I opened here, let’s say about 10 years, maybe more—I don’t even know anymore (a 36–45-year-old man), or since 2007, started trading (a 36–45-year-old woman). All the sellers who were interviewed (*n* = 18) unanimously confirmed that bear-themed souvenirs are consistently popular with tourists actively seeking them out. According to the sellers, tourists purchase these souvenirs for various reasons: As a memory. I think it associates the bear with the place. I also see on TV that there are many bears here (a 46–55-year-old man); we are known to have bears, that’s why (36–45-year-old woman). A variety of bear-themed souvenirs are available including postcards, mugs, toys, and small wooden sculptures, but the most prevalent choice among tourists is the fridge magnet (Figure 12): The first time there were pictures, then I brought postcards after the magnets. About 5 years ago, these carved ones also appeared (points to a bear’s head carved in wood) (a 36–45-year-old woman).

Usually, tourists who buy bear souvenirs are wild animal lovers (*n* = 14): I like all animals, in general. I grew up with bears in various forms, toys, drawings (a 46–55-year-old woman); yes, they are very nice (a 56–65-year-old man). Some (*n* = 3) were circumspect: I don’t know... (and he shakes his head in wonder) (man, over 65 years old); let’s say, they are beautiful in the forest, in the cities, aren’t they? (a 36–45-year-old woman) or “no, how can I like them? Do I like being eaten?” (a 36–45-year-old man, totally upset).

Tourists mostly want to buy souvenirs with bears. Fourteen interviewees strongly stated this, but others stated that they do not specifically look for souvenirs with bears: I do not specifically look for souvenirs with bears, but if I see something I like, I buy it (a 26–35-year-old woman). A few tourists (*n* = 3) indicated that they do not always purchase souvenirs or they having difficulty making a decision: They don’t always buy souvenirs (a 26–35-year-old man).

Memories with bears remain a positive association for families and close ones: I buy for myself (a 56–65-year-old-man); or for the family, I buy for family, but also for me; respectively for me and my family (a 36–45-year-old woman). That is why the most sought-after souvenirs are fridge magnets, which sellers unanimously agreed upon: If I find something, I like it. There are lots of bear magnets… have you seen them? Not sure what to choose! (an 18–25-year-old woman); to put it on the fridge, I always buy magnets. I have them there as a memory (a 26–35-year-old woman); I generally look for fridge magnets with the locations I go to. I’ll probably get one with bears too (a 56–65-year-old man).

## 4. Discussion

This study contributes to ongoing posthumanist discussions in geography emphasizing human–wildlife interactions. It specifically delves into human attitudes towards bears in shared habitats, seeking a mechanism for successful coexistence beyond the prevalent anthropocentric mindset. Local inhabitants expressed a heightened awareness and fear of bear encounters, with some implementing protective measures and associating bears with danger to human well-being and advocating for their preservation in the natural environment. On the other hand, tourists exhibited varied perspectives, with some considering the presence of bears as a potential threat that may reduce tourist numbers, while others view it as an opportunity to attract visitors.

### 4.1. Causes of the HBI and the Induced Risk

The bears’ migration to urban areas was not prompted by natural causes, such as drought [93], but rather the invasion of their habitat through deforestation for road construction, residential neighborhoods, and tourist infrastructure [17,32]. Additionally, forestry activities exacerbated the loss of habitat connectivity and the absence of compact forests across extensive areas [65].

Consistent with other studies conducted in Romania [94] and in Prahova Valley, the anthropogenic variable plays a decisive role in the incidence of HBIs and bear encounters. The presence of human food in containers and landfills acts as a lure and attracts brown bears to urban spaces [68]. Therefore, people should not feed bears because they will become accustomed to receiving food [65,95], increasing the frequency of bears entering human communities simultaneously with interspecies conflicts [12,22]. Following field investigations and personal observations, we found that the management of food and household waste are the intrinsic causes of the presence of bears in Prahova Valley [33]. Another cause is the food intentionally offered to bears by tourists to entice them to emerge for viewing [4,32] and for photos/films, and by local residents out of pity and compassion [34,66]. The intentional feeding of bears, both by locals and tourists, induces ambiguity in perceptions. Following these considerations, we observed that bears are constantly seeking food in an environment predominantly shaped by human presence. We can raise the question of whether this crowded environment is transforming into a landscape of food, as indicated by Brown et al. [96]. In this context, as some interviewees pointed out, feeding bears could still be evidence of a coexistence strategy and an understanding of posthumanist ideals in a shared landscape.

The field investigations highlighted a great danger and risk to the communities of Prahova Valley from the encroachment of the bear, losing its natural distance from people and potentially causing human casualties and direct damage [33]. The low percentage assigned to the absence of risk perhaps highlights the attachment of bears to the humans who feed them, but also that of humans to the bears through compassion. This reduced trans-species affinity in our case is still an attribute of ethical posthumanism, which does not exclude either compassion or violence determined by HBIs [46,97]. Compassion is a feeling of connection between humans and non-human animals, bears in this case, highlighting the lack of difference between species and the existential limitation of humans in a human-dominated landscape [46].

### 4.2. From Interaction to Coexistence through Adaptation

The Romanian press plays a significant role in shaping the narrative around human–bear interactions, with the local press in Prahova Valley [35] being part of the broader context within the country. The evidence of bear presence in tourist resorts instilled confidence among the inhabitants regarding the causes of risk exposure, prompting them to proactively learn and implement personal and group protective measures against bear encounters, as reported by the interviewees. It is interesting to mention that in Prahova Valley, as well as in the center of Romania [32], a large number of respondents had similar feelings of anxiety and fear regarding the presence of bears in their communities and imminent HBIs. On the other hand, humans develop fear and a sense of danger in their interaction with bears [98]. The fear of large carnivores (brown bears) influenced both attitudes and behaviors of people through avoidance or defense [67]. Therefore, there are restrictions of access to public spaces in the resorts at certain times of the day when bears are likely to be present, and these tactics vary based on personal protective strategies and willingness to take on risk, as inhabitants adjust their behavior [99]. On the other hand, some respondents showed forms of intolerance towards bears: bears were expected to live in their natural environment and not in cities, and residents accepted either the shooting or relocation of the bears. These control tactics are unlikely to happen, and coexistence still remains an alternative and viable strategy in the current context [32,86]. The bears in Prahova Valley live in an area dominated by humans and sometimes feel threatened by them. Avoiding contact with humans is their natural reaction [98], stemming from the fear of encountering them. Perhaps that is why humans are considered predators [96], and bears live in a landscape of fear [100,101]. This environment is characteristic of Prahova Valley due to the presence of human settlements, means of transportation, and economic as well as touristic activities, especially during the warm season between May and September. 

Conflicts are solved through coexistence, as the primary objective of conservation [47,102]. Therefore, conflict is not considered a part of coexistence; it is managed to a tolerable level [47,102]. However, we believe that in Prahova Valley the perception of residents and tourists in recent years highlights the beginnings of a peaceful coexistence that has surpassed the sometimes alarmist tone in the local press. Though less common, some respondents mentioned their emotional attachment to bears, similar to what happens, for example, in Spain [103]. This is an example of a posthuman relationship between humans and brown bears, with some respondents expressing care and compassion for bears, sentiments specified in the literature [104,105].

The local mass media initially induced fear, panic, and terror among residents and tourists by extensively reporting the presence of bears in various locations including streets, yards, in front of some schools, and even in cemeteries. The news also highlighted isolated attacks and injuries of some tourists (refer to Tables S2 and S4). Consequently, people’s perception of the HBI risk was amplified [70,71]. The imminent threat of risk can significantly influence people’s beliefs, attitudes, and behaviors [63].

The media can amplify people’s concerns about risk, even when it is low [63]. Subsequently, we observed a shift in the tone of mass media coverage (Table S3) that initially alarmed the public by announcing the places where the bear sought food such as dumpsters, restaurant terraces, or residential yards. Over time, this coverage evolved, losing its alarmist character and fostering sentiments against the presence of bears in tourist resorts. This shift towards tolerance, as reflected in the media reports, may represent the beginning of a posthumanist approach, aligning with the conservation efforts for large predators [66,67].

On the other hand, mass media releases that alarm the population about bears lowers the level of acceptance of the bears and influences bear management [79,106]. The responsiveness of decision-makers [103,107] and the public’s trust or distrust of them are key elements in risk management [69]. The residents of Prahova Valley have recently begun to trust information and safety programs advising how to prevent conflicts with bears [108,109] “for the facilitation of environmental awareness and future transformation of the environments we live”, as stated in [108]. Knowledge about bears, especially regarding their behavior and the way it is taken into account by decision-makers for coexistence management, is still interpreted from an anthropocentric perspective [99]. Effective coexistence management between humans and non-humans relies on local knowledge, community needs, and applied scientific measures [110,111] in a way that respects animal ethics in the spirit of posthumanism. Wildlife management policies, in the broad sense, are complex and depend on the realities of the local context [40,112]. Thus, the combination of “social, cultural, historical, biological, ecological, political, economic, and geographic components” makes each conflict or coexistence situation unique [111], and the local policies in place determine a specific mode of interaction between humans and non-humans.

As Toncheva [113] mentioned for the Rodopi Mountains in Bulgaria, in Prahova Valley the multitude of HBIs has led to the formation of behavioral responses on both sides, including awareness, avoidance, or peaceful reactions. Both bears and humans have developed patterns and signs of identification in the field [114]: the bear knows the location of garbage bins, establishes its routes in the city in search of food, and recognizes sheltering places during the day in parks and gardens. Meanwhile, humans are aware of the entry points of bears into the city, seal and protect garbage bins, and have developed personal practices to avoid encounters with bears.

We believe that a genuine guide for guidance and learning has been established, implemented in local memory, emphasizing coexistence strategies within a “landscape of tolerance” [98]. Consequently, as mentioned for other locations [58], in Prahova Valley, after many years of human–bear interaction experiences, an initial project was implemented (see Section 4.3.), which is familiar to those interviewed. We hope that this represents a first achievement in developing a harmonious coexistence, as an element of shared life between bears and humans in a landscape altered by humans.

### 4.3. Toponyms and Souvenirs, Attributes of the Opportunism of Tourist Attraction

Posthumanism is a reflective, ontological, and methodological vision worthy of consideration in tourism studies, as it modifies the ethical barriers of modern tourism from an anthropocentric perspective [112]. In a broader sense, Fennell and Sheppard [115] mention in a posthumanist conceptual framework that ecotourism can better address non-human entities by enhancing the well-being and rights of animals.

Toponyms highlight the existence of bears as a natural ecological factor [116,117]. In Prahova Valley, the toponyms reflect the bear’s habitat, with steep, rocky and rugged slopes [36,118] specific to the Bucegi Mountains.

Numerous elements of the tourist infrastructure emerged in the area following the Romanian Revolution of 1989 to enhance its appeal to tourists. This choice of location was intentional, as the main traffic route DN1 connects Bucharest, the capital of Romania, to Brașov, the country’s primary tourist city, situated on the outskirts of the forest and in close proximity to deforested areas where the bears enter the resorts [34]. The opportunistic behavior of local tourism professionals should be interpreted within the context of the post-communist reclamation of the bear image as idyllic. The use of bear-related names for tourist establishments serves as a symbol of local identity, generating interest and attracting tourists.

Souvenirs play an important role in social memory, preserving past experiences, fostering tourists’ inclination for return visits, and serving as a source of place identity [119,120]. The bear has become an important tourist attraction in Prahova Valley, ranging from its symbolic representation to its physical presence. Street vending of souvenirs is simultaneously a way to increase the socio-economic value of wildlife, in this case, the brown bear, while also providing support for conservation efforts [121]. Souvenirs represent memories of the experience, and connect the tourist to the experience of the place [122,123]. As our surveys showed, postcards and fridge magnets are the most accessible souvenirs, which create links between people and places [124]. Not all tourists buy bear souvenirs, demonstrating an anti-tourist attitude [125]. Most of the interviewees answered unequivocally that they buy souvenirs with bears to have a memory of the destination that symbolizes the experience of their own trip [24]. This tourist shopping phenomenon is defined as the intensification experience [126]. The memories are retained within the family and among those close to them, setting off the retreat experience as narrow and intimate [127].

The bear, as a non-human entity, contributes to the formation of tourist experiences. These experiences are materialized through the sale of souvenirs featuring various representations of bears, representing an anthropocentric activity that contradicts the idea of posthumanism. The immediate economic profits of souvenir sellers benefit tourism, often disregarding the rights of non-human animals [40,128], leading to their marginalization [129].

Uncertainty remains about whether tourism policy will progress in a manner that takes into account the well-being and rights of both human and non-human entities together. Nevertheless, it is argued that the pursuit of equal rights for all beings is the only trajectory, specifically the path of ethical posthumanism, that we must aim to follow [130].

## 5. Conclusions

This study underlines the complex dynamics and varied perceptions surrounding HBIs, with implications for tourism, conservation, and community safety in Prahova Valley. The novelty of this study is based on both its theoretical foundation and its integrative methodological approach, using a diverse range of qualitative and quantitative information within a posthumanist perspective, beyond anthropocentric interests. Through this methodology, we could delve deeper into the study area to discover more profound elements in HBIs. Beyond these considerations, this method can be easily applied in other studies in the field of human–animal interaction (for example, with dogs, cats, elephants, and more), and is not limited to just HBIs.

The narratives used in the analysis were based on concrete facts, telling the story of the place and holding special significance for both residents and tourists. Many HBI experiences were reflected in the communication of narratives, from which ambivalent information was extracted regarding the perceptions of locals and tourists during encounters with bears.

Despite being contested, we argue that the sale of souvenirs, a relatively recent activity, will continue to generate a permissive framework for tolerance, coexisting through adaptation between humans and bears, and a better understanding of interactions.

The question remains whether, in the current context, the landscape of fear or the landscape of food, based on valuable elements of the complex human–bear interaction in Prahova Valley, will foster debates and practical implementations that surpass anthropocentric rigidity and encourage the increasingly evident emergence of posthumanist thinking.

We consider that we have addressed the two questions. Firstly, in Prahova Valley, within a human-modified landscape, there are encouraging signs of a harmonious coexistence between humans and bears, represented by feelings of compassion, tolerance, and acceptance towards the bears. Secondly, the success of this coexistence is evident due to the opening up to posthumanism, as a portion of the residents and tourists in the Prahova Valley has started to ethically think beyond strictly human, biased, anthropocentric interests, and to seriously consider the interests and rights of other non-human animals. If mass media promoted fear, terror, panic, and imminent attacks after HBIs, later the news took on a shade of posthumanist ethics. It is noteworthy in this regard the trend to overcome the ancestral fear of bears, the diminishing idea of physical suppression of bears, the initiation of projects for peaceful coexistence, and the awareness and education of the local population, as well as the emotional overcoming of alarmist news distributed in the media.

Furthermore, in Prahova Valley, various strategies for conserving the bear’s habitat, along with management and educational programs, have been implemented to mitigate HBIs. These initiatives are crucial for raising awareness among the Prahova Valley population, addressing both bear behavior and the importance of initiating safety measures for fostering coexistence between humans and bears.

## Data Availability

The data presented in this study are available on request from the corresponding author. The data are not publicly available due to the fact that there is a huge volume of information resulted from interviews and surveys that need further translation from the Romanian Language.

## Supplementary Materials

The following supporting information can be downloaded at https://www.mdpi.com/article/10.3390/ani14040587/s1. Table S1: (a) Characteristics of the questionnaires; (b) Characteristics of the interviews; (c) Characteristics of the selected mass media, and (d) Characteristics of souvenirs sellers and buyers; Table S2: The location of brown bears in the local press; Table S3: Reports in the local mass media about bears in search of human food, and Table S4: Human fear related to bear encounters as reflected in the mass-media headlines.
