# Supplementary material for: Living with Bears in Prahova Valley, Romania: An Integrative Analysis
Source: Animals (Basel). 2024 Feb 10;14(4):587. doi: 10.3390/ani14040587 (PMC10885976; doi:10.3390/ani14040587)
Supplement: Supplementary file 1 [file animals-14-00587-s001.zip › Table S1.pdf]

Characteristics of the questionnaires

a. Survey				
Period	Sample			Number of questions
1 June-1 July 2021	210			24
August 2022	Gender	Age	Employment status	21 closed
	67% female<br>33% male	43.9% between 18 and 25 years<br>30.7% between 26 and 35 years<br>12.2% between 36 and 45 years<br>11.2% between 46 and 55 years<br>2% over 55 years	employee 138 (66.3%)<br>students 63 (30.7%)<br>unemployed 6 (2.4)<br>retired 3 (0.5%)	2 open<br>one that uses a Likert scale

Characteristics of the interviews

b. Interviews				
Period	Sample			Employment status
1 July-1 August 2022	36 people			
	Age groups	Male	Female	
	9 between 26 and 35 years<br>11 between 36 and 45 years<br>9 between 46 and 55 years<br>7 over 55 years	19	17	4 taxi and uber driver<br>3 meteorology<br>3 cable car workers<br>3 administrators of the tourist guesthouse<br>2 members of Sinaia and Buşteni MRPS<br>6 employee<br>10 tourists<br>1 head of hunting association<br>2 ski monitor<br>1 PhD in geography<br>1 Director of Bucegi Natural Park

Characteristics of the selected mass media

c. Mass media				
Newspaper or newspaper platform	Newspaper type and territorial distribution	Periodicity of the newspaper	General policy of the newspaper	No. of selected news items/date of news publication
Adevărul.ro	general news newspaper/national	daily	center-left	3/2020; 4/2021; 5/2022
Romaniatv.net	multimedia/national platform	daily	center-left	4/2022
Ziare.com	multimedia/national platform	daily	center	1/2022
Observatornews.ro	multimedia/national platform	daily	center	4/2017; 5/2018; 7/2019; 8/2021; 4/2022
Rebublikanews.ro	multimedia/national platform	daily	center	1/2021
Știrileprotv.ro	multimedia/national platform	daily	right/liberal orientation	1/2021
Prahovei Valley Tv	multimedia/regional platform	daily	center	1/2018

Characteristics of souvenirs sellers and buyers

d. Souvenirs			
Period:10-25 April			
Sample 1			Status
age groups	male	female	
4 between 26 and 35 years<br>6 between 36 and 45 years<br>6 between 46 and 55 years<br>2 over 55 years	10	14	sellers
Sample 2			Status
age groups	male	female	
4 between 26 and 35 years<br>7 between 36 and 45 years<br>3 between 46 and 55 years<br>3 over 55 years	16	12	tourists
