# Supplementary material for: Living with Bears in Prahova Valley, Romania: An Integrative Analysis
Source: Animals (Basel). 2024 Feb 10;14(4):587. doi: 10.3390/ani14040587 (PMC10885976; doi:10.3390/ani14040587)
Supplement: Supplementary file 1 [file animals-14-00587-s001.zip › Table S2.pdf]

Date	Newspapers	News
12.11.2018	observatornews.ro	Unusual images in a neighbourhood in Buşteni. Two bears tried to get into a car.
16.05.2019	observatornews.ro	Buşteni has become the favourite resort of bears.
13.07.2019	https://a1.ro	Tourist attacked by a bear in the centre of Predeal resort. The man was saved by a dog.
14.07.2019	observatornews.ro	Tourist attacked by a bear in Predeal. The man escaped after a struggle with the wild animal.
15.06.2020	observatornews.ro	The bears have moved to the resorts in the Prahova Valley.
16.06.2020	Adevărul.ro	Dozens of bears chased away recently from the centre of the mountain resorts in Prahova.
20.12.2020	observatornews.ro	The bears scared the tourists in Sinaia.
18.03.2021	Adevărul.ro	A bear and her cub, on an evening walk through the courtyards of Azuga.
21.03.2021	Adevărul.ro	“Safari" in the Prahova Valley. The tourists who rode the Sinaia Express train were assisted from the side by two bear cubs.
28.05.2021	rebublikanews.ro	Bears, increasingly present in Prahova Valley.
03.02.2022	observatornews.ro	"See, she came after you!" Bear and her cub, chased away with snowballs, at Peleş Castle.
9.06.2022	Adevărul.ro	The bears ended up walking like cats in the parking lots of the blocks in Buşteni. Images captured in the middle of the day.
09.06.2022	romaniatv.net	Bears walk in the streets of the Prahova Valley like in the forest. The locals regard them as pets, and the authorities have forgotten to intervene. Incredible pictures!
04.07.2022	observatornews.ro	Panic in Buşteni. A bear with a cub was seen rummaging through a dumpster in front of a house.
18.07.2022	ziare.com	The resort is full of tourists while a bear roams the streets unhindered.
21.07.2022	romaniatv.net	The bears are preparing for the weekend.
28.11.2022	romaniatv.net	Caravan torn apart by bears in Bucegi Mountains. Viral images on TikTok.
