# Supplementary material for: Living with Bears in Prahova Valley, Romania: An Integrative Analysis
Source: Animals (Basel). 2024 Feb 10;14(4):587. doi: 10.3390/ani14040587 (PMC10885976; doi:10.3390/ani14040587)
Supplement: Supplementary file 1 [file animals-14-00587-s001.zip › Table S3.pdf]

Date	Newspapers	News
17.11.2017	https://observatornews.ro	Bears in the resorts of the Prahova Valley, a problem without solutions. Some locals would intentionally leave them food.
24.11.2018	observatornews.ro	Hunger took the bears out of the forest again. A female, together with three cubs, went out on the streets of Buşteni, looking for food.
27.11.2018	https://observatornews.ro	Bears, a nocturnal visit to a neighbourhood in Buşteni. A female with three cubs was caught looking for food in dumpsters.
20.04.2019	https://observatornews.ro	The bears came down to Bușteni and rummaged through the dumpsters of the locals.
18.01.2021	știrileprotv.ro	Bears filmed when they jump the fence and enter a household in Buşteni.
21.03.2021	https://evz.ro	Panic at a restaurant in Predeal.
10.05.2021	https://replicaonline.ro	A bear was filmed while looking for food on a street in Sinaia.
02.06.2021	https://observatornews.ro	A hungry bear cub entered a yard in Sinaia. In search of "breakfast", the animal overturned a dumpster.
02.06.2022	https://observatornews.ro	A hungry bear cub rushed into a yard in Sinaia. In search of "breakfast", the animal overturned a dumpster.
06.06.2022	https://adevarul.ro	A bear entered a guesthouse in Predeal.
