# Supplementary material for: Living with Bears in Prahova Valley, Romania: An Integrative Analysis
Source: Animals (Basel). 2024 Feb 10;14(4):587. doi: 10.3390/ani14040587 (PMC10885976; doi:10.3390/ani14040587)
Supplement: Supplementary file 1 [file animals-14-00587-s001.zip › Table S4.pdf]

Date	Newspapers	News
14.11.2017	observatornews.ro	Eight bears were seen on the streets of Sinaia, Buşteni, and Azuga resorts. Man attacked, rushed to hospital. The gendarmes intervened.
15.11.2017	observatornews.ro	In Sinaia, the authorities intervene with force, every day, because of the INVASION OF BEARS. The locals are desperate and afraid to leave their homes.
09.04.2019	observatornews.ro	A hungry bear entered a clinic in Azuga. There were several patients and employees of the institution in the yard.
16.08.2019	observatornews.ro	Two bears caused panic in a gas station in Azuga.
01.09.2019	observatornews.ro	Seven bears caused panic in Cerbului Valley from Buşteni.
17.01.2021	observatornews.ro	Panic in Azuga: Bears in search of food have also arrived in people's yards.
14.03.2021	Adevărul.ro	Bear filmed breaking the fence of a household in Azuga.
15.06.2021	observatornews.ro	A bear caught while chasing two people in Sinaia. People had no idea what was happening behind them.
07.07.2021	observatornews.ro	Bear filmed entering a household in Sinaia. People are terrorized and ask for help: Bears and wild boars condemn us to a life full of fears.
22.08.2021	observatornews.ro	Two bears were walking unhindered on the streets of Buşteni. A woman walking her dog bumped into them.
25.09.2021	observatornews.ro	Man attacked and mauled by a bear right behind the house in the Prahova Valley. The dogs jumped to defend him: "He will remain maimed for the rest of his life".
26.09.2021	observatornews.ro	Two men barely escaped with their lives after an encounter with the bears in the Prahova Valley: "Out of the desire to protect animals, we forgot to take care of people".
04.11.2021	Adevărul.ro	Young woman from Sinaia chased by bears in a resort alley. The images prove the nightmare that the locals in the mountain areas go through.
4.07.2022	observatornews.ro	Panic in Buşteni. A bear with a cub was seen rummaging through a dumpster in front of a house.
13.07.2022	Adevărul.ro	Locals from the Buşteni resort, terrorized by bears.
13.09.2022	Adevărul.ro	Incredible images captured at Buşteni. Three bears climb a metal fence with almost human gestures.
01.11.2022	https://www.gandul.ro	A bear went for a walk in Predeal, just a few metres from a park where children and parents were playing.
12.11.2022	romaniatv.net	A bear rushed into a cemetery.
23.11.2022	observatornews.ro	Panic in Azuga after a bear appeared in front of the school. The children were in class.
